# Supplementary material for: BayesAge: A maximum likelihood algorithm to predict epigenetic age
Source: Front Bioinform. 2024 Apr 4;4:1329144. doi: 10.3389/fbinf.2024.1329144 (PMC11024280; doi:10.3389/fbinf.2024.1329144)
Supplement: Supplementary file 1 [file DataSheet2.PDF]

Supplementary Material

1 SUPPLEMENTARY DATA

We include the traits data with subject names and ages with the data *Traits_HumanMultiTissue458.csv*. We uploaded the main data with the methylation matrix along with ages (*sample_458_age.csv*), the data with the methylated counts (*CountMatrix-M_HumanMultiTissue458_20-100.csv*) and the total counts (*CountMatrix-N_HumanMultiTissue458_20-100.csv*) under the `data` folder on the official GitHub page. The thymine count can be calculated by subtracting the total counts by the methylated counts.

2 SUPPLEMENTARY TABLES AND FIGURES

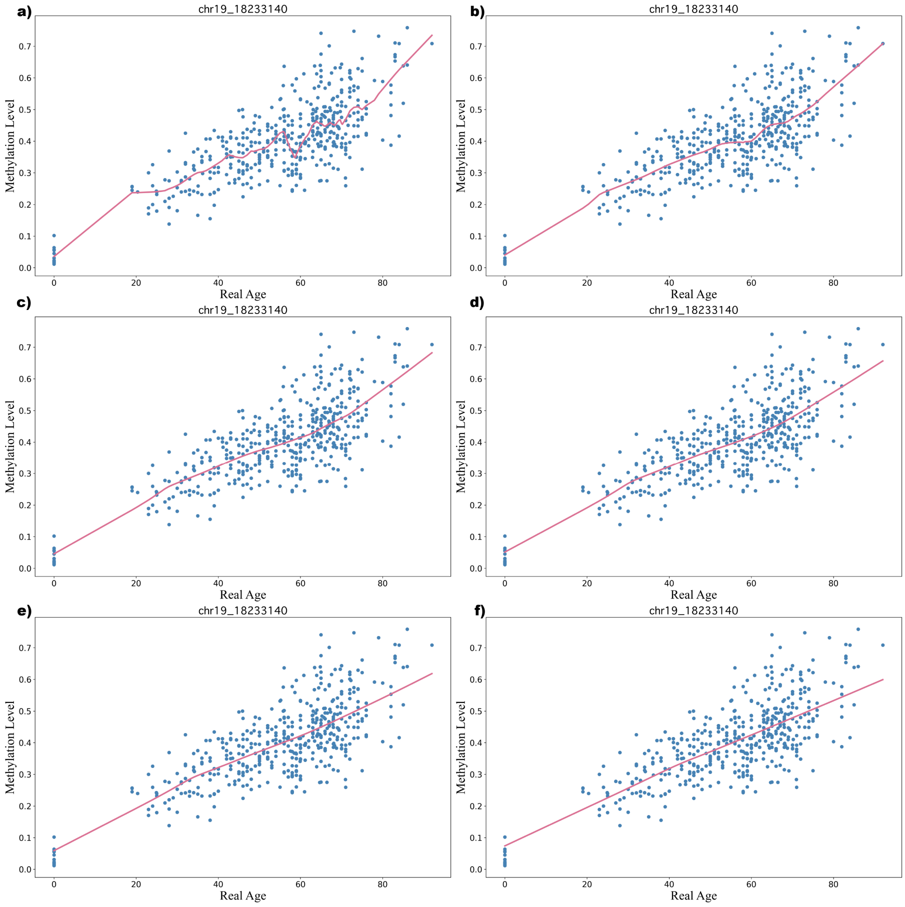

Figure S1: How LOWESS regression fits with different tau values for a random top cpg site *chr19_18233140*. a) has a τ value of 0.1. b) has a τ value of 0.3. c) has a τ value of 0.5. d) has a τ value of 0.7. e) has a τ value of 0.9.f) has a τ value of 1.0.

The table below shows the r-squared values and the mean absolute error of the predicted ages using lowess fits with different tau values without downsampling.

Tau Value	R-squared	MAE
0.1	78.7%	6.86
0.3	78.6%	6.98
0.5	78.4 %	6.99
0.7	78.0%	7.00
0.9	77.4%	7.18
1.0	77.4 %	7.15

Table S1. R-squared and MAE of age predictions using different tau values.
