# Supplementary figures and images for: BayesAge: A maximum likelihood algorithm to predict epigenetic age
Source: Front Bioinform. 2024 Apr 4;4:1329144. doi: 10.3389/fbinf.2024.1329144 (PMC11024280; doi:10.3389/fbinf.2024.1329144)

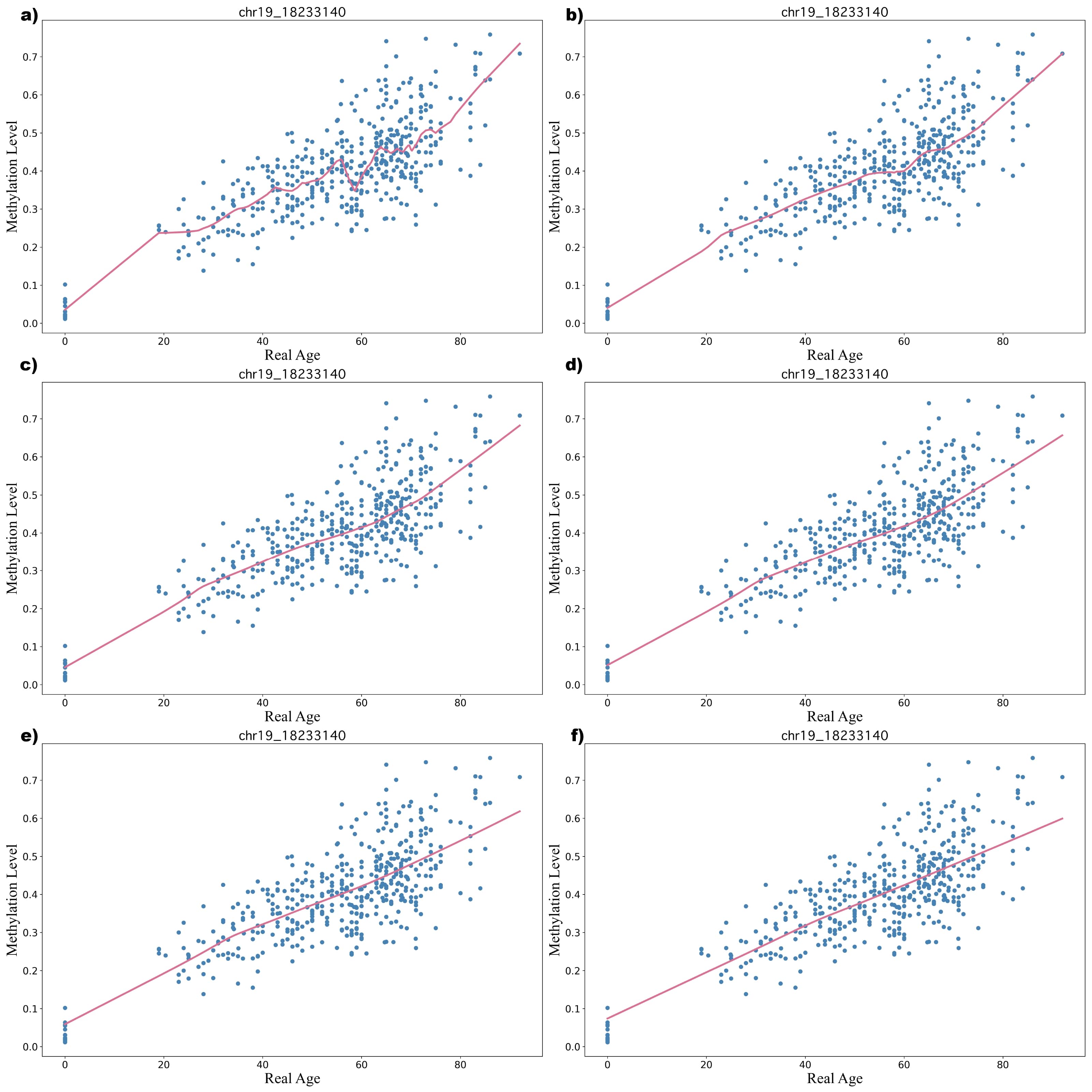

Supplement: Supplementary file 2 [file Image1.JPEG]
